# Comparison of safety and immunogenicity between Healive®, Havrix® and live attenuated Hepatitis A vaccines in pediatric population: a systematic review with meta-analysis

**DOI:** 10.1186/s13052-025-01996-8

**Published:** 2025-12-02

**Authors:** Mohamed Abo Zeid, Amr Elrosasy, Mohamad Ali Farho, Mohamed Rifai, Menna M. Aboelkhier, Mohamed Nabil Elkhrashy, Ahmed Hamdy Zabady, Mohamed Samir A. Zaki, Eman Mostafa Hamed, Samy A. Dawood, Yasmine Abuzaid

**Affiliations:** 1 Faculty of Medicine Tanta University, Tanta, Egypt; 2 Faculty of Medicine, Cairo University, Cairo, Egypt; 3 Faculty of Medicine, University of Aleppo, Aleppo, Syrian Arab Republic; 4 Faculty of Medicine Menofyia University, Shebin El Kom, Egypt; 5 Master Program, Faculty of Science, Cairo University, Cairo, Egypt; 6 Faculty of Medicine, Al-Azhar University, Cairo, Egypt; 7 Department of Microbiology, Faculty of Science, Damanhour University, Damanhour, Egypt; 8 Department of Anatomy, College of Medicine, King Khalid University, Abha, Saudi Arabia; 9 Department of Clinical Pharmacy, Faculty of Pharmacy, Galala University, Suez, Egypt; 10 Department of Child Health, College of Medicine, King Khalid University, Abha, Saudi Arabia

**Keywords:** Hepatitis A, Healive vaccine, Inactivated vaccine, Live attenuated vaccine, Havrix

## Abstract

**Background:**

Healive is an inactivated vaccine for hepatitis A virus developed in China and was found to be well-tolerated and highly immunogenic in adults and children. It is our aim this study to compare the safety and immunogenicity of Healive® with Havrix® and live attenuated vaccines in pediatric populations.

**Methods:**

A systematic search of PubMed, Scopus, Web of Science, and Embase databases was conducted following PRISMA guidelines. Randomized controlled trials (RCTs) comparing Healive with other vaccines were included.

**Results:**

Seven RCTs involving 3664 patients were included. Healive showed comparable efficacy to Havrix regarding seroconversion rates and GMT at one month (SMD = 0.24, 95% CI [-0.11 to 0.59]), but achieved better results at six (SMD = 0.85, 95% CI [0.57 to 1.07]) and seven months (SMD = 0.55, 95% CI [0.41 to 0.70]). When compared to live attenuated vaccines, Healive demonstrated superior GMT at one month (SMD = 0.31, 95% CI [0.07 to 0.56]) and two years (SMD = 0.36, 95% CI [0.06 to 0.67]).

**Conclusion:**

Healive appears to be effective and safe for preventing hepatitis A, providing at least five years of protection. This review underscores the importance of ongoing research to optimize hepatitis A vaccination strategies, including standardized assays for antigen content, clarification of protective antibody levels, and large-scale trials in regions of intermediate endemicity.

**Supplementary Information:**

The online version contains supplementary material available at 10.1186/s13052-025-01996-8.

## Impact statement

- Healive is an effective and safe hepatitis A vaccine, offering comparable or superior efficacy to other vaccines, with protection lasting at least five years.
- This study provides a comprehensive comparison of Healive’s performance against Havrix and live attenuated vaccines, emphasizing its stronger GMT at longer follow-up periods.
- The findings support Healive as a reliable option for hepatitis A prevention, guiding vaccination strategies and future research.

## Background

Hepatitis A, one of the largest health care problems globally, is an acute infection of the liver induced by the hepatotropic hepatitis A virus (HAV). HAV is the most prevalent form of acute viral hepatitis worldwide [1, 2].

Hepatitis A is an enteral viral infection caused by the HAV, a single-stranded RNA-virus. HAV is mainly transmitted by the fecal–oral route in areas with poor sanitation, thus contaminated food and water, person-to-person contact, and men having sex with men (MSM), can all transmit this virus. Very rarely infection occurs parenterally via blood products. In addition to improving hygiene, particularly in water supplies and sewage disposal, there has been a decrease in natural immunity to the disease [1, 3, 4].

The prevalence of HAV antibodies in the population varied from 15% to almost 100% in various regions of the world [3].

Regarding the clinical presentation of HAV infection, it is usually benign in young children, but it can cause evident disease in adults and adolescents, ranging from mild anicteric infection (anorexia, malaise, fatigue, abdominal discomfort, vomiting and diarrhea) to fulminant liver failure, usually necessitating liver transplantation. Disease severity and case-fatality are strongly correlated with age. Most deaths are caused by fulminant hepatic failure. Infection-induced immunity persists for life [3, 5–7].

There are no effective drugs for the treatment of HAV so far. Vaccination is an effective measure to provide immunization against HAV infection and reduce its incidence. Since the first commercial hepatitis A vaccine was licensed in Switzerland in 1991 [8, 9] It has proven to be effective in inducing anti-HAV seroprotection in many clinical trials [10].

With the development of the economy and the improvement of hygienic measures, more people have no natural immunity to HAV infection, resulting in a higher likelihood for outbreaks of hepatitis A. More hepatitis A vaccine should be provided to prevent the potential outbreak in a vulnerable population [3, 11].

The World Health Organization (WHO) recommends routine immunization programs in countries where a large proportion of the adult population is susceptible to HAV. Immunization might also eliminate the major source of infection for other children by cross contamination; and ultimately prevent infection in the older age groups as the immunity provided by HAV vaccines is long-lasting [12].

Two types of vaccines (live attenuated vaccine and inactivated vaccine) are available. Numerous hepatitis A vaccines have been introduced in the market after successful isolation of HAV in 1979. Broadly, there are 2 types of hepatitis A vaccines available globally; formaldehyde inactivated vaccines (usually used) and live attenuated vaccines (manufactured in China) [13, 14].

Many studies have been published about inactivated, well accepted, and acknowledged, vaccines in the international literature. The live attenuated H2 strain vaccine (H2 vaccine) was licensed for human use in 1992. Apart from its country of origin, China, is now available in many countries. The vaccine is available by the brand name of Zhepu in China and Biovac-A (Wockhardt Ltd.) in India. While this vaccine has proven to be safe and effective even with a single dose and supported with many short term and long-term studies, much of the data is either scattered or published in Chinese [14–17].

In 2001, a new preservative-free inactivated hepatitis A vaccine Healive® was developed by Sinovac Biotech in China. According to both pre-approved and post-approved trials, Healive® was safe, well-tolerated and highly immunogenic in adults and children [18, 19].

It is our aim in this review to evaluate the immunogenicity and safety of Healive comparing it with other vaccines like Havrix and Hepatitis A live attenuated vaccines.

## Materials and methods

The procedural framework of this investigation adhered to the methodology outlined in the Cochrane Handbook for Systematic Reviews and Meta-analysis [20]. We followed the PRISMA statement guidelines [21] in reporting this meta-analysis. The whole study was registered on PROSPERO under number [CRD42024514271].

### Literature search

We conducted a methodical search across the subsequent databases: PubMed, Scopus, Web of Science (WOS), and Embase, aiming to retrieve relevant published studies from their inception until February 2024. We used keywords to build our search strategy including.

“Healive” OR"HAV vaccine” and “HAV” OR"hepatitis A virus” OR “hepatitis”.

In adhering to our thorough approach, we conducted a manual search of the reference lists of studies meeting our initial criteria to guarantee the comprehensive inclusion of potentially relevant studies and to minimize the possibility of missing any pertinent literature during our search process. All duplicates were removed by Endnote software. Additional details can be accessed in Table 1***.***

**Table 1:** Details of search strategy among databases

Database	Keywords	NO
*PubMed*	*(Healive OR"HAV vaccine") AND (HAV OR"hepatitis A virus"OR hepatitis)*	**260**
*Scopus*	*Healive OR"HAV vaccine"AND HAV OR"hepatitis A virus"OR hepatitis*	**338**
*WOS*	*Healive OR"HAV vaccine"AND HAV OR"hepatitis A virus"OR hepatitis*	**236**
*Embase*	*Healive OR"HAV vaccine"AND HAV OR"hepatitis A virus"OR hepatitis*	**357**

Rayyan software [22] was used during the selection process; two reviewers independently and blindly assessed the retrieved references in a two-stage process: initially screening titles and abstracts of all extracted articles, followed by a second phase involving a comprehensive full-text screening of all eligible abstracts. In case of any discrepancies, a third reviewer helped in resolving the conflicts.

### Selection and eligibility criteria

In the process of selecting relevant studies, we adhered to a specific set of criteria outlined by PICO. In this investigation, *participants*: we took into consideration both healthy and diseased patients. *Intervention*: our focus was mainly on Healive® vaccine other than others.

*Comparison*: we selected the related studies that have placebo, Harvix vaccines, or other biological vaccines. *Outcome*: Our main outcomes included a set of key interns; primary outcomes through this investigation were efficacy which can be measured through both Seroconversion rate (SR) and geometry mean titer or concentration (GMT or GMC) within follow-up periods beginning with 1 month till 60 months (5 years). Besides, we considered adverse events which might be associated with the secondary outcomes. *Study design*: we focused on studies with prospective follow-up like RCTs and prospective observational studies.

We excluded non-English studies, case-reports, animal studies, reviews, editorials, studies with only an abstract or unavailable full text or overlapped data…etc.., and studies with other intervention or without a comparison group also will be excluded.

### Data extraction

Data from eligible studies was gathered on a standardized sheet for data extraction form by two independent reviewers. Then a cross-verification was conducted, and any discrepancies were addressed through discussion. The uniform data extraction sheet encompasses three domains, from which details related to the included studies are derived, first domain was: *characteristics of included studies* such as (study ID, Study design, Country, Number of registrations in case of RCTs, centers’ numbers, study arms, route of vaccine administration, method of vaccine preparation, sample size, and follow-up duration). The second domain was: *participants characteristics* at *baseline* including: (Age, gender, Height, and weight), furthermore, the third domain included the outcomes that we highlighted on them previously.

### Risk of bias assessment

Two independently blinded investigators, employing the Cochrane Collaboration’s Risk of Bias tool (version 2) [23], evaluated the quality of the incorporated studies across the primary domains as following: randomization process, deviation from the intended interventions, missing outcome data, measurement of the outcome, and selection of the reported result, five primary categories that make up the composite score would be used by this tool. The investigators’ judgments are classified as"Low risk,""Some concerns,"or"High risk"of bias for each of these topics. The summary and graph of the RoB2 tool were generated on Review Manager Software (RevMan, version 5.4 for Windows) [24].

A third investigator reanalyzed the conflicts and rectified them. The funnel plot indicating publication bias couldn’t be established as the number of included studies were minimized.

### Statistical analysis

For comprehensive analysis of extracted data, we used RevMan, version 5.4 software tool provided by Cochrane collaboration for analysis and construction of forest blots; for outcomes reported as continuous outcomes, we pooled them as standardized mean difference (SMD). For dichotomous outcomes, we pooled them as Risk ratio (RR) and their corresponding confidence interval (CI) using the Mantel–Haenszel method, we also performed subgroup analysis based on the duration of follow-up.

Additionally, for assessment of statistical heterogeneity among included studies, a visual inspection of the forest plots was used, also the Chi-square test (known as the Cochrane Q test) and Higgns and Thompson *I*^2^ which has the following formula *I*^*2*^ = *((Q-df)/Q)* × *100%.* were used to quantify it. Based on, if the value of *I*^2^ would exceed more than 50% and the chi-square test’s *p*-value was less than 0.1, the statistical heterogeneity would be considered to be significant between studies, and *DerSimonian Laird random effects models* would be used. These models are effective in resolving the heterogeneity between studies.

On the other hand, heterogeneity would be fluctuated as low, moderate, and high whether.

*I*^2^valued as < 25%, from 25–75%, or > 75%, respectively [25].

Meta-regression was performed using OpenMeta v5.26.14 software to explore any cause of heterogeneity and to evaluate if there is any correlation between seroconversion rates and the age of administration or the incidence of adverse events between the examined vaccines with a significance level set at *p* < 0.05.

## Results

### Search results and study selection

**Fig. 1:**
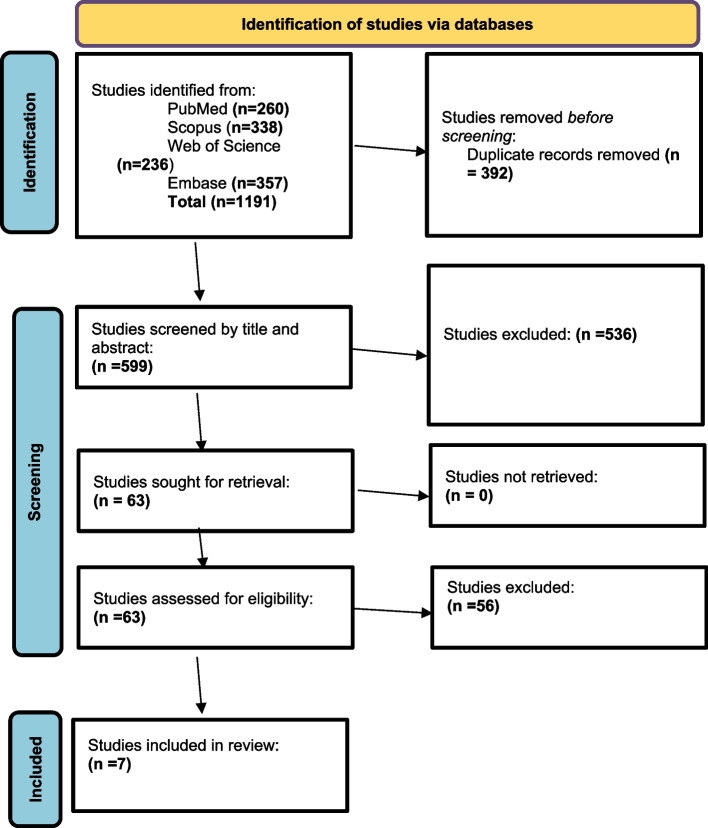
PRISMA flow chart

On applying the predetermined search strategy, 1191 articles were retrieved. After the elimination of 392 duplicated studies, 536 further papers were excluded throughout the screening process for titles and abstracts. 56 more studies were excluded throughout the full-text screening process. In the end, seven articles [26–32] were deemed eligible for inclusion in this systematic review’s quantitative and qualitative synthesis (Fig. 1).

### Characteristics of the included studies

Seven randomized controlled trials (RCTs) [26–32] were incorporated into our analysis compromising 3664 patients that received either inactivated hepatitis A vaccine (Healive (HAPIBEV) or Havrix) or live attenuated hepatitis A vaccines. Only one study was conducted in India while the remaining six studies were conducted in China, with a follow up duration ranging from seven days to 60 months.

The mean age of patients was ranging from 3.1 to 7.5 years while the mean height was ranging from 94.8 to 123.9 cm, and the mean weight was ranging from 14.9 to 23.7 kg (Table 2 and 3).

**Table 2:** Summary of included studies

Study ID (author-year)	Country (No. of Centers)	Study Design	Sample size (Follow-up duration)	Study Arms	Primary Outcomes	Main inclusion criteria	Vaccine preparation & administration	Conclusion
**Zheng 2011 ** [31]	China (Two-centers)	RCT	841 (1- Month)	-Hepatitis A vaccine (H2 strain) -Healive® vaccine -Havrix® vaccine -Hepatitis B vaccine	Measurement of anti-HAV positive rate and GMC among 4 types of vaccines over 28 days	-An axillary temperature ≤ 37 ◦C -Normal ALT test -Negative HBsAg and anti- HAV tests	-Group A: one dose of freeze dried live attenuated hepatitis A Vaccine referred to H2 vaccine -Group B: one dose of domestic inactivated hepatitis A vaccine (Healive®, 250 UI) -Group C: one dose of imported inactivated hepatitis A vaccine (Havrix®, 720 EU) Administration: intramuscular injection into the deltoid region using auto disable syringes	The study was not able to identify differences between Havrix®, Healive® and H2 vaccines in terms of seroconversion proportion and GMC* between seven and 28 days
**Zhang 2012 ** [29]	China (Single center)	RCT	303 (6-Months)	-Healive®vaccine -Havrix® vaccine	comparison interchangeability and safety of Healive and Havrix among Chinese children	-An axillary temperature ≤ 37 ◦C -Normal ALT test -Negative HBsAg and anti-HAV tests	Healive: 1-Hepatitis A virus strain TZ84 was cultivated in 2BS human fetal lung diploid fibroblast by cell factory technology 2-The virus was harvested, purified by chromatography, inactivated by formalin 3- adsorbed onto aluminium hydroxide. A 0.5 mL-dose contained 250 u antigen and 0.25 mg alum Havrix (0.5 ml/dose) contained 720 ELISA units (El. U) antigen and 0.25 mg alum Administration: intramuscular injection into the deltoid region	The present study indicated that both vaccines can be recommended for interchangeable using of immunization among Chinese healthy children
**Liu 2013 ** [27]	China (multi-center)	RCT	841 (12- Months)	-Hepatitis A vaccine (Healive®) with a one-dose or two-dose schedule -one-dose of one of the three kinds of live attenuated	vaccines SRs and GMCs of anti-HAV IgG in the 5 vaccination groups, At 6- and 12-months- follow-ups	-Children age 1.5–6 years -Informed written consents should be obtained from parents or guardians before inclusion of their children in the study	-The inactivated hepatitis A vaccine (Healive®): pediatric formulation with 250 U/ml of hepatitis A antigen Administration: intramuscular injection into the deltoid region -The inactivated hepatitis A vaccine (Healive®) and three RCT kinds of live tenuated vaccine^a^ stored at 4° C	A higher GMC* of anti-HaV IgG was induced in the two-dose Healive® than in the one-dose and the attenuated vaccines at 12 months The attenuated vaccine B or C produced higher GMCs* than the one-dose Healive® at 6–12 months after vaccination
**Liu 2015 ** [28]	China (multi-center)	RCT	202 (36- Months)	-Healive® vaccine with one-dose or two-dose schedule −1 dose of the live attenuated vaccine (Biovac)	concentration of antibody to hepatitis A virus [Time Frame: 36 months]	-Healthy undergraduate students -Aged 16 to 25 years -Sign the informed consent -Provide an ID	- Healive: inactivated hepatitis A vaccine was formulated for individuals aged ≥ 16 years, contained 500 U/mL hepatitis A virus (HAV) antigen Administration: intramuscular injection into the deltoid region - Administration: Attenuated vaccines with a titer of 6.5 log 50% cell culture infective doses (CCID50) were given subcutaneously in the deltoid region -The inactivated and attenuated vaccines were stored at 4 °C, with the cold chain maintained during transport	-High rates of sero-protection persisted for at least 36 months among adults who received one or two doses of inactivated hepatitis A vaccine among adults than who received one dose of live attenuated vaccine -The long-term monitoring of immunogenicity induced by one dose of inactivated hepatitis A vaccine is needed to determine an effective alternative to a 2-dose schedule
**Yu 2016 ** [26]	China (Single center)	RCT	375 (66-Months)	-Healive® vaccine -Havrix® vaccine	comparison between GMCs and SRs of anti-HAV antibodies in the 2 groups at each timepoint from 1 to 66 month	-Aged 1—8 years -Healthy conditions as determined by history talking and physical examination, -Negative serum anti-HAV	1-Hepatitis A virus strain TZ84 was cultivated in 2BS human fetal lung diploid fibroblast by cell factory technology 2-The virus was harvested, purified by chromatography, inactivated by formalin 3- adsorbed onto aluminium hydroxide. A 0.5 mL-dose contained 250 u antigen and 0.25 mg alum, without preservative The control vaccine (0.5 mL/dose) contained 720 ELISA units (El. U) antigen and 0.25 mg alum -Both Healive® and the control vaccine were licensed products in China and stored at 2—8 ◦C Administration: The vaccines were administered intramuscularly into deltoid region	Compared with Harvix, the new preservative-free inactivated hepatitis A vaccine (Healive®) in 2 doses showed better persistence of antibody concentrations for 5 years after full-course immunization among children and the persistence of protective immunogenicity was estimated for at least 20 years
**Zhang 2017 ** [30]	China (Single center)	RCT	332 (5-Years)	- Healive® vaccine -Biovac ™ (live attenuated vaccine)	Determination of the 5-year persistence of antibodies to HAV in Chinese children after one dose of Chinese domestic	-Healthy children aged 18–60 months -pre-vaccination anti-HAV antibody titers < 20 mIU/ml	-(Group B) Healive: one dose of inactivated HA vaccine (Containing 250-unit hepatitis A antigen) -Group A: one dose of live attenuated HA vaccine (containing 6.50 lgCCID_50_ hepatitis A antigen, Biovac™)	-No significant difference in antibody persistence between 2 groups was found -No clinical hepatitis A case was reported -A single dose of an inactivated or live attenuated HA vaccine at [18–60 months] of age resulted in high HAV SR* and anti-HAV antibody concentrations that lasted for at least 5 years
**Thuluva 2021 ** [32]	India (Multicenter)	RCT	520 (210 days)	-HAPIBEV™ (Healive) -Havrix® vaccine	SRs and IgG GMCs and 2- and 4-Fold increase in IgG Antibody Concentration	Healthy and HAV vaccine-naïve children of either gender, who were 1–15 years of age	Each single pediatric dose of 0.5 mL of HAPIBEV vaccine contained inactivated HAV antigen (250U) in an adjuvant of aluminum hydroxide to be given as IM dosage	HAPIBEVTM vaccine demonstrated an immunological and safety profile on par with Havrix in 1–15 years old healthy HAV vaccine-naive Indian children

GMC*: Geometric mean concentrations

SR*: Sero-protection rates

^**a**^N. B: The inactivated and three live attenuated hepatitis A vaccines used in this clinical trial are licensed by State food and drug administration (SFDA, China) for routine use in humans

**Table 3:** Baseline demographic characteristics of included studies

Study ID (author-year)	Study arms	Age (years)^a^	Male/Female^b^	Height (Cm)^a^	Weight (Kg)^a^
Zheng 2011 [31]	**Healive®**	**7.5 (1.1)**	**109/99 (52%/48%)**	**123.9 (10.0)**	**23.23 (5.17)**
	**Havrix®**	**7.2 (1.1)**	**134/80 (63%/34%)**	**122.2 (9. 7)**	**22.74 (5.55)**
	**H2 vaccine (Live Attenuated Vaccine)**	**7.3 (1.2)**	**105/99 (51%/49%)**	**123.7 (9.3)**	**23.57 (5.97)**
Zhang 2012 [29]	**Healive® (2 doses)**	**4.08 (1.02)**	**40/36 (52.63%/47.37%)**	**103.7 (8.6)**	**18.0 (4.4)**
	**Havrix® (2 doses)**	**4.21 (0.91)**	**43/33 (56.58%/43.42%)**	**105.5 (7.3)**	**18.2 (3.3)**
Liu 2013 [27]	**Inactivated vaccine 2 doses**	**3.175 (1.24)**	**91/79 (53.5%/46.5%)**	**95.5 (11.5)**	**14.9 (3.2)**
	**Inactivated vaccine 1 dose**	**3.217 (1.15)**	**92/77 (54.4%/45.6%)**	**96.1 (12.5)**	**15.3 (6.7)**
	**Attenuated vaccine A 1 dose**	**3.38 (1.33)**	**83/89 (48.3%/51.7%)**	**97.0 (12.5)**	**15.3 (3.5)**
Liu 2015 [28]	**Inactivated Vaccine 2 Dose**	**NR**^a^	**NR**^a^	**NR**^a^	**NR**^a^
	**Inactivated Vaccine 1 Dose**				
	**Live Attenuated Vaccine**				
Yu 2016 [26]	**Healive® vaccine**	**3.8 (1.72)**	**142/141 (50.2%/49.8%)**	**100.7 (12.87)**	**17.2 (3.86)**
	**Havrix® vaccine**	**3.7 (1.47)**	**48/44 (52.2%/47.8%)**	**100.6 (12.97)**	**17.4 (3.91)**
Zheng 2017 [30]	**Inactivated HA vaccine**	**2.89 (0.36)**	**NR**^a^	**95.4 (6.6)**	**14.9 (2.9)**
	**Attenuated HA vaccine**	**2.89 (0.45)**	**NR**^a^	**96.5 (8.6)**	**15.6 (3.0)**
Thuluva 2021 [32]	**HAPIBEV**	**7.0 (3.85)**	**143/117 (55%/45%)**	**119.1 (23.90)**	**23.5 (11.73)**
	**Havrix**	**7.2 (3.80)**	**125/135 (48.08%/51.92%)**	**119.8 (23.98)**	**23.7 (11.79)**

*NR* Data were unreported

^a^Age, Height, Weight reported as Mean (SD)

^b^Gender reported as n (%)

### Quality assessment of the included studies

**Fig. 2:**
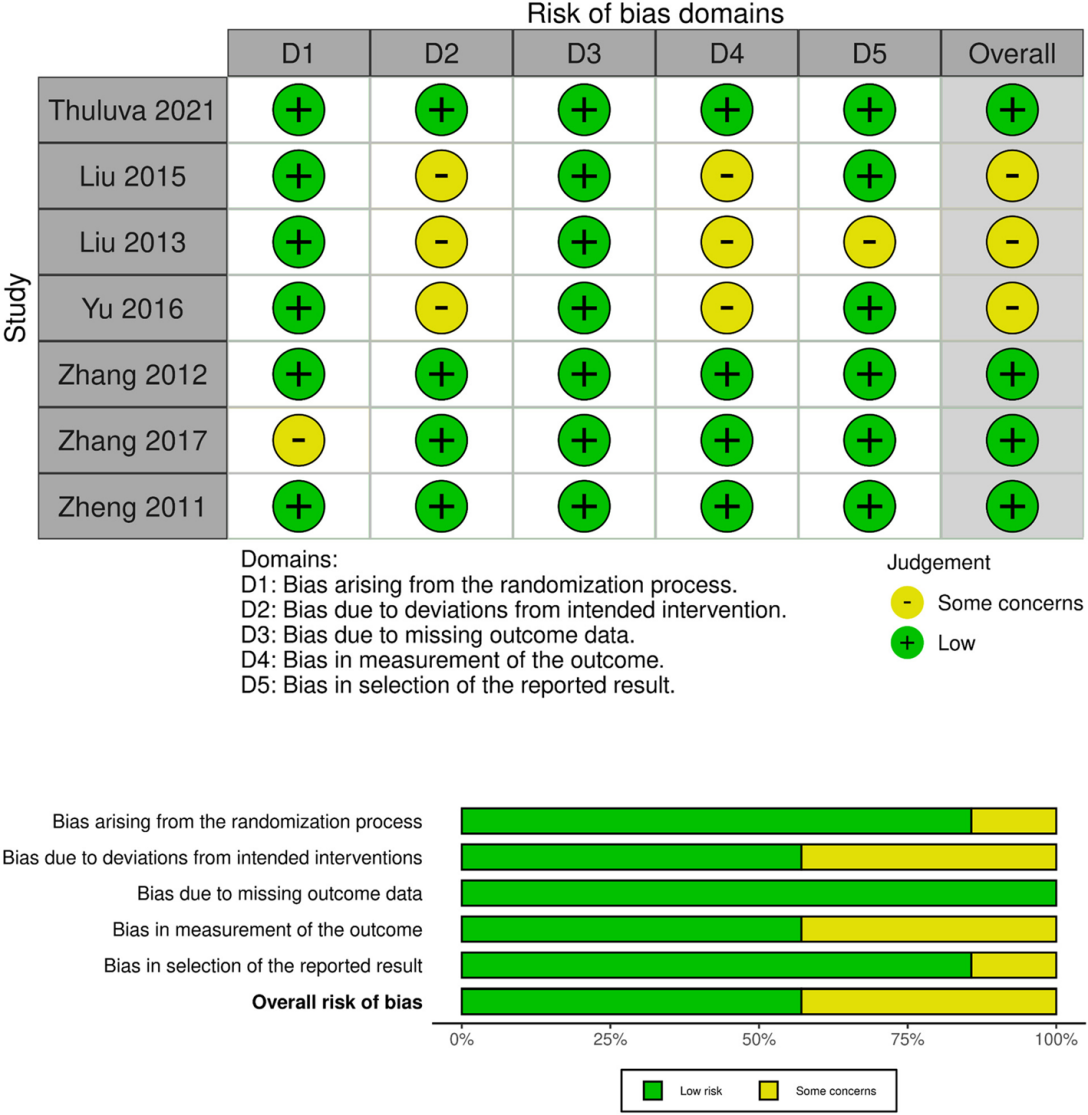
Summary of assessment of risk of bias

According to the Cochrane Risk of Bias Assessment Tool for Randomized Clinical Trials II (ROB-II), The quality of three studies [26–28] showed some concerns, while the remaining four studies [29–32] showed low risk of bias (Fig. 2).

### Healive vs Havrix

#### Immunogenicity

**Fig. 3:**
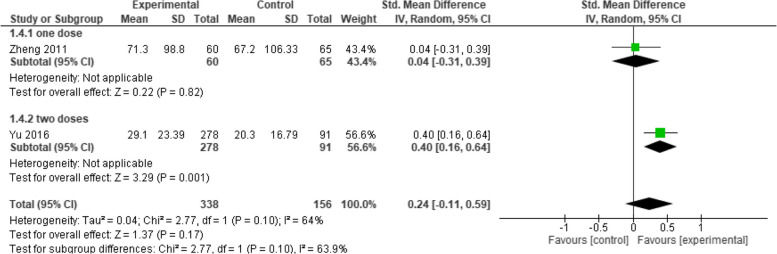
IgG GMC at one month (Healive vs Havrix). Comparison of IgG GMC between Healive and Havrix at one month (SMD = 0.24, 95% CI [−0.11 to 0.59])

**Fig. 4:**
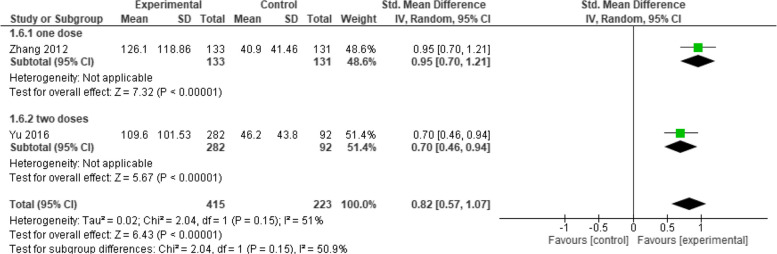
IgG GMC at six months (Healive vs Havrix). Healive showed higher IgG GMC than Havrix at six months (SMD = 0.85, 95% CI [0.57 to 1.07])

**Fig. 5:**
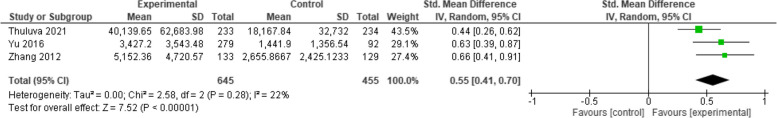
IgG GMC at seven months (Healive vs Havrix). Healive had higher IgG GMC than Havrix at seven months (SMD = 0.55, 95% CI [0.41 to 0.70])

When evaluating the IgG GMC between Healive and Havrix at one month, the overall mean difference between the two groups did not favor either of them (SMD = 0.24, 95% CI [−0.11 to 0.59], *p* = 0.17), with moderate heterogeneity among the pooled studies (*p* = 0.10, I2 = 64%) (Fig. 3). On the other, at six and seven months it was found that Healive achieved better GMC results than Havrix (SMD = 0.85, 95% CI [0.57 to 1.07], *p* < 0.00001) and (SMD = 0.55, 95% CI [0.41 to 0.70], *p* < 0.00001) respectively, with moderate heterogeneity found at six months (*p* = 0.15, I2 = 51%) (Fig. 4) and low heterogeneity at seven months (*p* = 0.86, I2 = 0%) (Fig. 5).

**Fig. 6:**
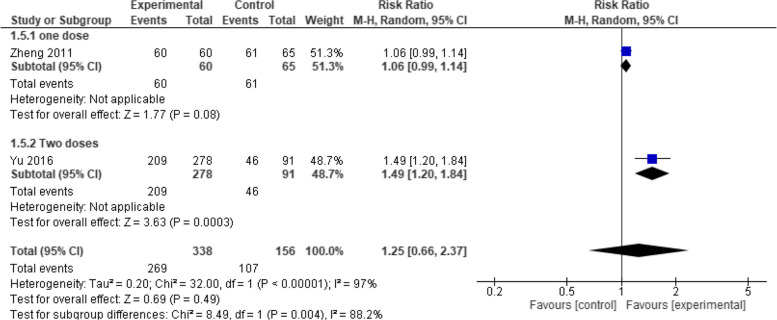
Seroconversion at one month (Healive vs Havrix). No significant difference in seroconversion between Healive and Havrix at one month (RR = 1.25, 95% CI [0.66 to 2.37])

**Fig. 7:**
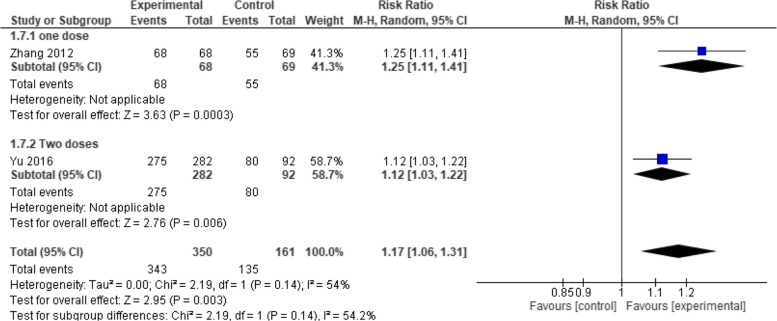
Seroconversion at six months (Healive vs Havrix). Healive had better seroconversion rates than Havrix at six months (RR = 1.17, 95% CI [1.06 to 1.31])

**Fig. 8:**
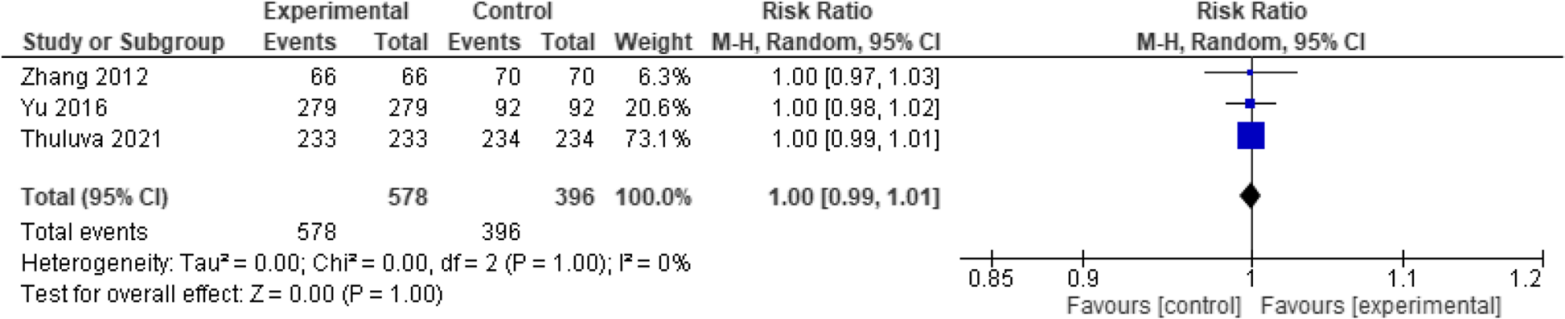
Seroconversion at seven months (Healive vs Havrix). No significant difference in seroconversion between Healive and Havrix at seven months (RR = 1.00, 95% CI [0.99 to 1.01])

Moreover, when evaluated at one month, seroconversion rate showed no significant difference between the two vaccines (RR = 1.25, 95% CI [0.66 to 2.37], *p* = 0.49) with high heterogeneity (*p* = 0.49, I2 = 97%) (Fig. 6). But when evaluated at six months, Healive achieved significantly better seroconversion rates than Havrix (RR = 1.17, 95% CI [1.06 to 1.31], *p* = 0.003) with moderate heterogeneity (*p* = 0.14, I2 = 54.2%) (Fig. 7). Finally, when evaluated at seven months, no significant difference was observed between the two vaccines (RR = 1.00, 95% CI [0.99 to 1.01], *p* = 1) with low heterogeneity (*p* = 1.00, I2 = 0%) (Fig. 8).

##### Meta-regression results

Meta-regression analysis was conducted between the two vaccines in seroconversion rate after adjusting for age and the rate of adverse events to determine if any pediatric groups were at low risk of developing adverse events, and we found no significant difference between the two groups: age (*p* = 0.732) and adverse events (*p* = 0.764). (Supplementary Fig. 1 and 2).

#### Safety

##### Total adverse events

**Fig. 9:**
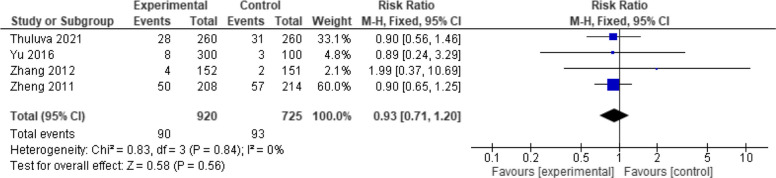
Total adverse events (Healive vs Havrix). No significant difference in total adverse events between Healive and Havrix (RR = 0.93, 95% CI [0.71 to 1.20])

Four studies were included in this outcome with 1645 patients. The overall effect estimates showed a non-significant difference between the two vaccines (RR = 0.93, 95% CI: [0.71 to 1.20], *p* = 0.56). The pooled results showed no heterogeneity (*p* = 0.84, I2 = 0%) (Fig. 9).

##### Vomiting

**Fig. 10:**
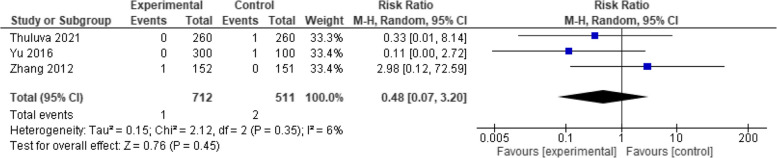
Vomiting (Healive vs Havrix). No significant difference in vomiting between Healive and Havrix (RR = 0.48, 95% CI [0.07 to 3.20])

The analysis of two studies with 703 patients showed a non-significant difference in overall effect estimates (RR = 0.48, 95% CI [0.07 to 3.20], *p* = 0.45 and low heterogeneity (*p* = 0.35, I2 = 6%) (Fig. 10).

##### Fever

**Fig. 11:**
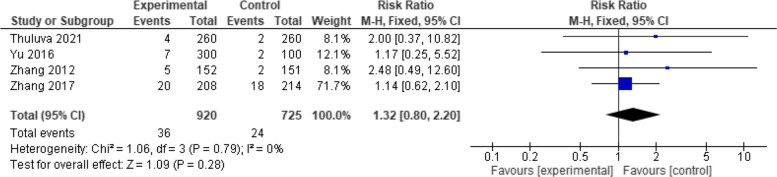
Fever (Healive vs Havrix). No significant difference in fever between Healive and Havrix (RR = 1.32, 95% CI [0.80 to 2.20])

On analyzing 1645 patients in four studies, the overall effect estimates demonstrated a non-significant difference between the two vaccines (RR = 1.32, 95% CI: [0.80, 2.20], *p* = 0.28). The pooing results showed homogeneity (*p* = 0.79, I2 = 0%) (Fig. 11).

### Healive vs live attenuated

#### Immunogenicity

**Fig. 12:**
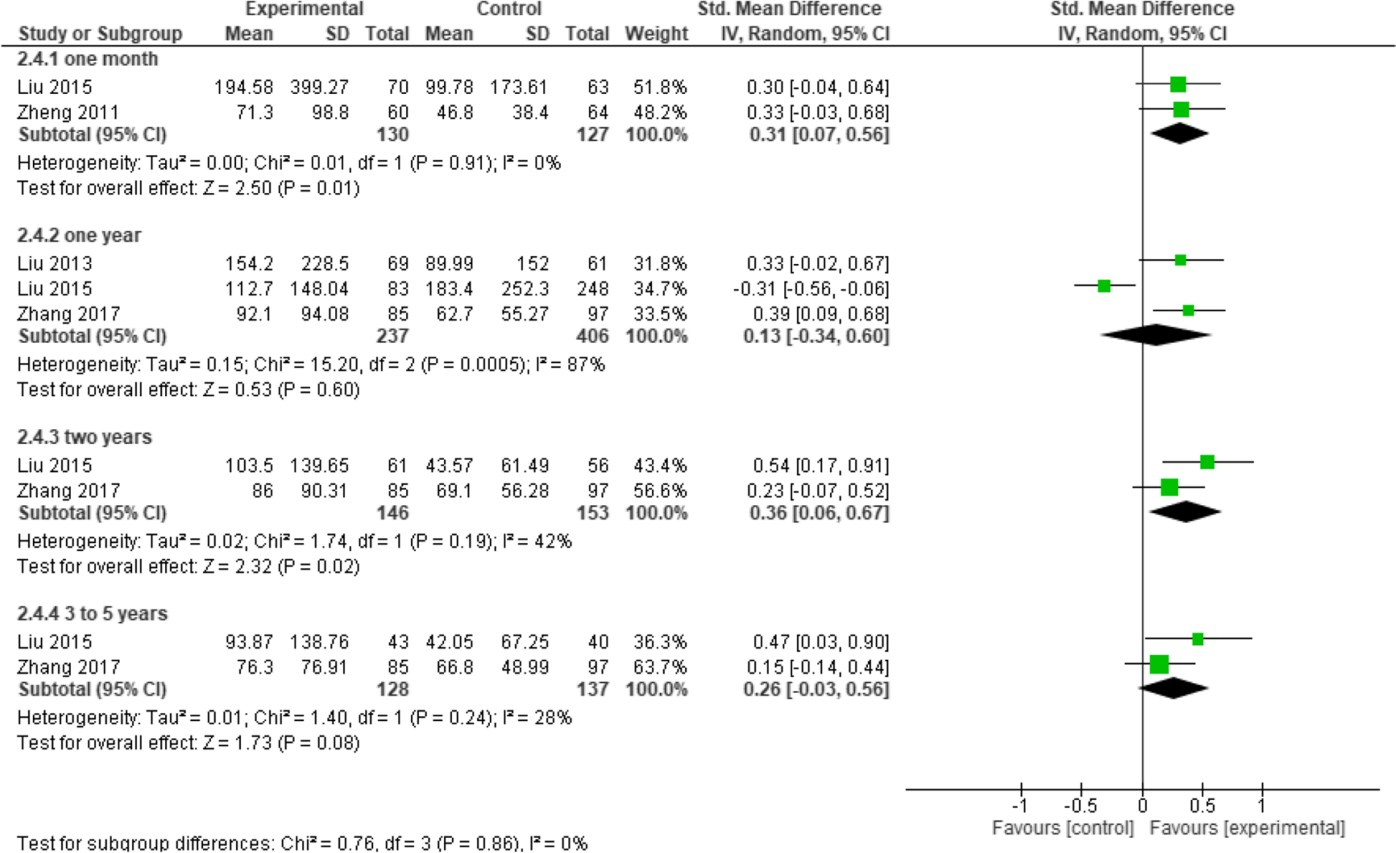
IgG GMC (Healive vs Live attenuated). Healive had higher IgG GMC than live attenuated vaccine at one month (SMD = 0.31, 95% CI [0.07 to 0.56]) and two years (SMD = 0.36, 95% CI [0.06 to 0.67])

On evaluating IgG GMC between the two groups subgroup analysis was performed and showed that the overall effect estimates favored Healive over the live attenuated vaccine at one month and two years of administration (SMD = 0.31, 95% CI: [0.07 to 0.56], *p* = 0.01) and (SMD = 0.36, 95% CI: [0.06 to 0.67], *p* = 0.02) respectively, but no significant results were observed between the two groups at one year and from three to five years (SMD = 0.13, 95% CI: [−0.34 to 0.60], *p* = 0.60) and (SMD = 0.26, 95% CI: [−0.03 to 0.56], *p* = 0.08) respectively (Fig. 12).

**Fig. 13:**
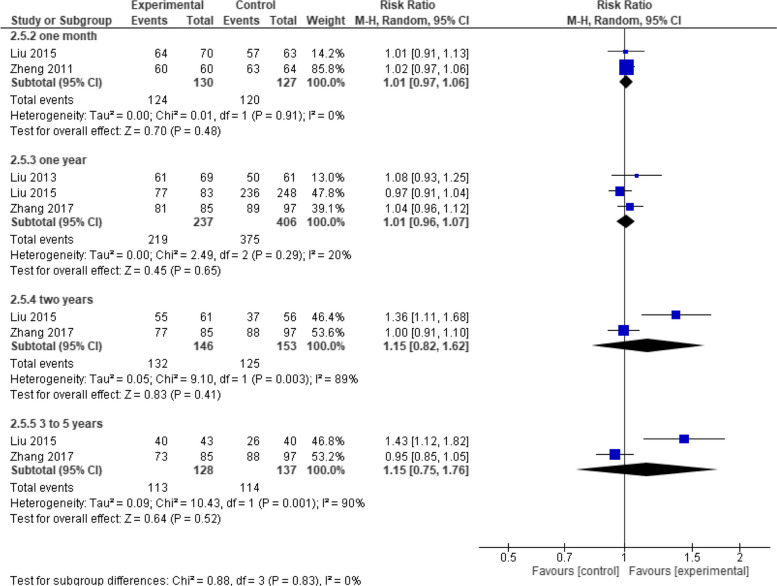
Seroconversion (Healive vs Live attenuated). No significant difference in seroconversion between Healive and live attenuated vaccine at any time point

While on evaluating the seroconversion rates we performed subgroup analysis across the different periods of examination and it showed no significant results between the two groups at one month, one year, two years and at five years (RR = 1.01, 95% CI [0.97 to 1.06], *p* = 0.48), (RR = 1.01, 95% CI [0.96 to 1.07], *p* = 0.65), (RR = 1.15, 95% CI [0.82 to 1.62], *p* = 0.41), (RR = 1.15, 95% CI [0.75 to 1.76], *p* = 0.52) respectively (Fig. 13).

#### Meta-regression results

Meta-regression analysis was conducted between the two vaccines in seroconversion rate after adjusting for age and the rate of adverse events to determine if any pediatric groups were at low risk of developing adverse events, and we found no significant difference between the two groups: age (*p* = 0.468) and adverse events (*p* = 0.413) (Supplementary Fig. 3 and 4).

#### Safety

##### Total adverse events

**Fig. 14:**
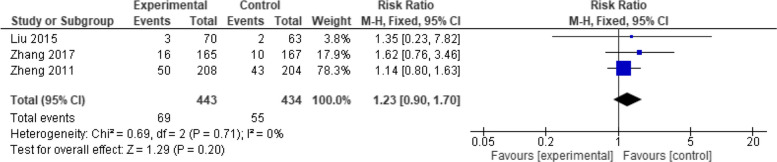
Total adverse events (Healive vs Live attenuated). No significant difference in total adverse events between Healive and live attenuated vaccine (RR = 1.23, 95% CI [0.90 to 1.70])

Three studies were included in this outcome with 877 patients. The overall effect estimates showed a non-significant difference between the two vaccines (RR = 1.23, 95% CI: [0.90 to 1.70], *p* = 0.20). The pooled results showed no heterogeneity (*p* = 0.71, I2 = 0%) (Fig. 14).

##### Fever

**Fig. 15:**
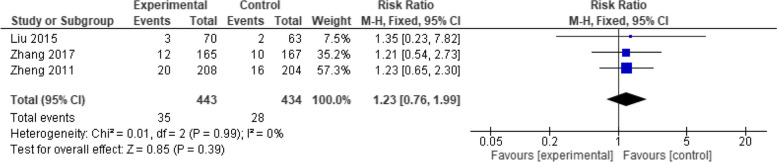
Fever (Healive vs Live attenuated). No significant difference in fever between Healive and live attenuated vaccine (RR = 1.23, 95% CI [0.76 to 1.99])

Analyzing 877 patients in three studies, the overall effect estimates demonstrated a non-significant difference between the two vaccines (RR = 1.23, 95% CI: [0.76 to 1.99], *p* = 0.39). The pooled results maintained homogeneity (*p* = 0.99, I2 = 0%) (Fig. 15).

## Discussion

To the best of our knowledge, this is the first meta-analysis to compare between Healive with other vaccines for preventing hepatitis A. Healive and Havrix were evaluated. The overall SMD did not favor either vaccine at one month, but Healive achieved better GMC results at six and seven months. Healive also achieved significantly better seroconversion rates at six months. The overall effect estimates showed a significant difference favoring Healive over the live attenuated vaccine regarding GMC results. However, no significant results were observed between the two groups at one and from three to five years. Seroconversion rates were also not significantly different between the two groups. No significant difference was noted for local or serious adverse events compared to Havrix or live attenuated vaccine.

The current review includes three systematic reviews on the effectiveness, safety, cost-effectiveness, and impact of hepatitis A vaccines. The first review, a 2012 Cochrane review, found insufficient evidence to support the use of Healive for preventing hepatitis A and recommended further studies. The results suggest that vaccination with the inactivated HAV vaccine significantly affects conferring sero-protective anti-HAV IgG [33]. The second review, an Ott 2012 review, found that protective anti-HAV virus antibody levels after inactivated vaccine can persist for almost 11 years and increase or reappear after booster vaccination. However, further research is needed on the vaccine doses needed for long-term protection against hepatitis A infection [34]. The third review, a 2022 SAGE Hepatitis A vaccines systematic review, found that hepatitis A vaccines are effective in preventing HAV clinical disease and confer seroprotection, regardless of the type of vaccine (live attenuated or inactivated). They also confer long-term protection against hepatitis A related diseases, including seroprotection [35]. Our regression analyses indicated no significant relationship between age, AEs and seroconversion rates for either vaccine. These findings align with previous studies that have shown hepatitis A vaccines to be well-tolerated across different age groups in the pediatric population [36].

Even though our searches were comprehensive, we could identify only a small number of randomized trials. The trials varied in design, country settings, populations, treatment regimens, follow up periods, and comparator. There was a lack of direct comparisons of single vs two dose HAV vaccine regimens over long-term periods. The WU 2023 study was observational, and AEFIs may be coincidental symptoms of vaccination or psychogenic reactions not related to vaccination [36]. Most studies focused on clinical disease effectiveness and impact evaluation, with no assessment of other relevant outcomes like death and hospitalization rates. Some evidence was found through booster challenges, but these studies were small-scale.

The study adhered to the Cochrane Handbook of Systematic Reviews, followed PRISMA guidelines, searched multiple electronic databases, included prospective controlled trials, except one retrospective cohort study, and conducted the review using a published, peer-reviewed protocol.

The Inactivated hepatitis A vaccine (Healive) significantly reduces the risk of contracting hepatitis A in susceptible individuals, providing at least 5 years of protection, and this review provided evidence to support the safety of the inactivated HAV vaccine.

This review suggests several key recommendations for future research on the inactivated HAV vaccine. It suggests the development of a standard assay to compare antigen content between manufacturers, following ICH-GCP’s definition of adverse events, well-designed randomized clinical trials with comparative designs, and high-quality post-marketing surveillance. It also calls for clarification on the absolute protective level of anti-HAV, particularly on levels less than 10 mIU/ml. Large, well-constructed trials are needed in regions of intermediate endemicity. Further investigation is needed into the duration of protection following a single dose of vaccine. Future trials should consider reporting patients with hepatitis by time since vaccination, use cluster randomization, and adhere to CONSORT Guidelines.

## Supplementary Information

Supplementary Material 1

## Data Availability

The datasets used and/or analyzed during the current study are available from the corresponding author on reasonable request.
