# Supplementary material for: Comparison of safety and immunogenicity between Healive®, Havrix® and live attenuated Hepatitis A vaccines in pediatric population: a systematic review with meta-analysis
Source: Ital J Pediatr. 2025 Dec 2;51:316. doi: 10.1186/s13052-025-01996-8 (PMC12690888; doi:10.1186/s13052-025-01996-8)

**Supplementary figure 1:** Regression plot for AEs and seroconversion rate between Healive® and Havrix®. This figure shows the relationship between AEs and seroconversion rate for the Healive® vaccine, with the Omnibus p-value = 0.764.


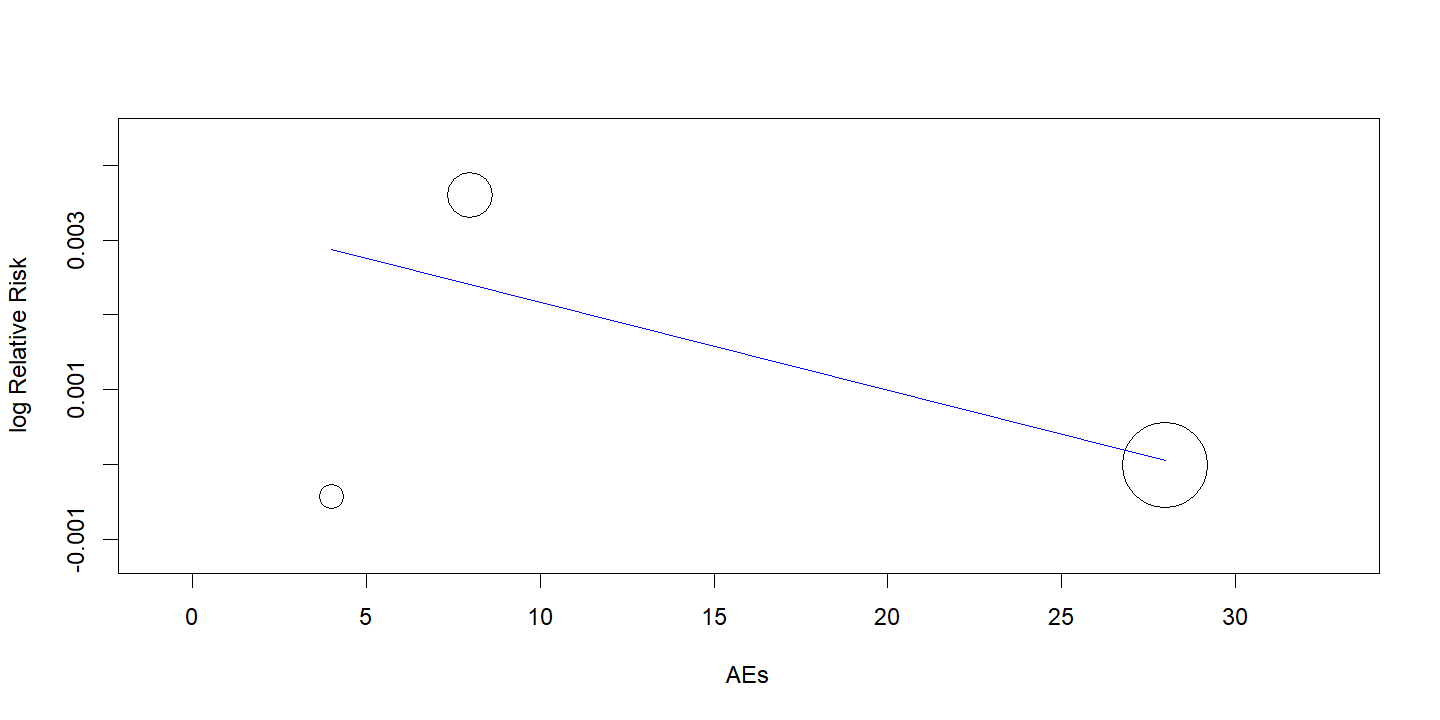


**Supplementary figure 2:** Regression plot for age and seroconversion rate between Healive® and Havrix®. This figure shows the relationship between age and seroconversion rate for the Healive® vaccine, with the Omnibus p-value = 0.732.


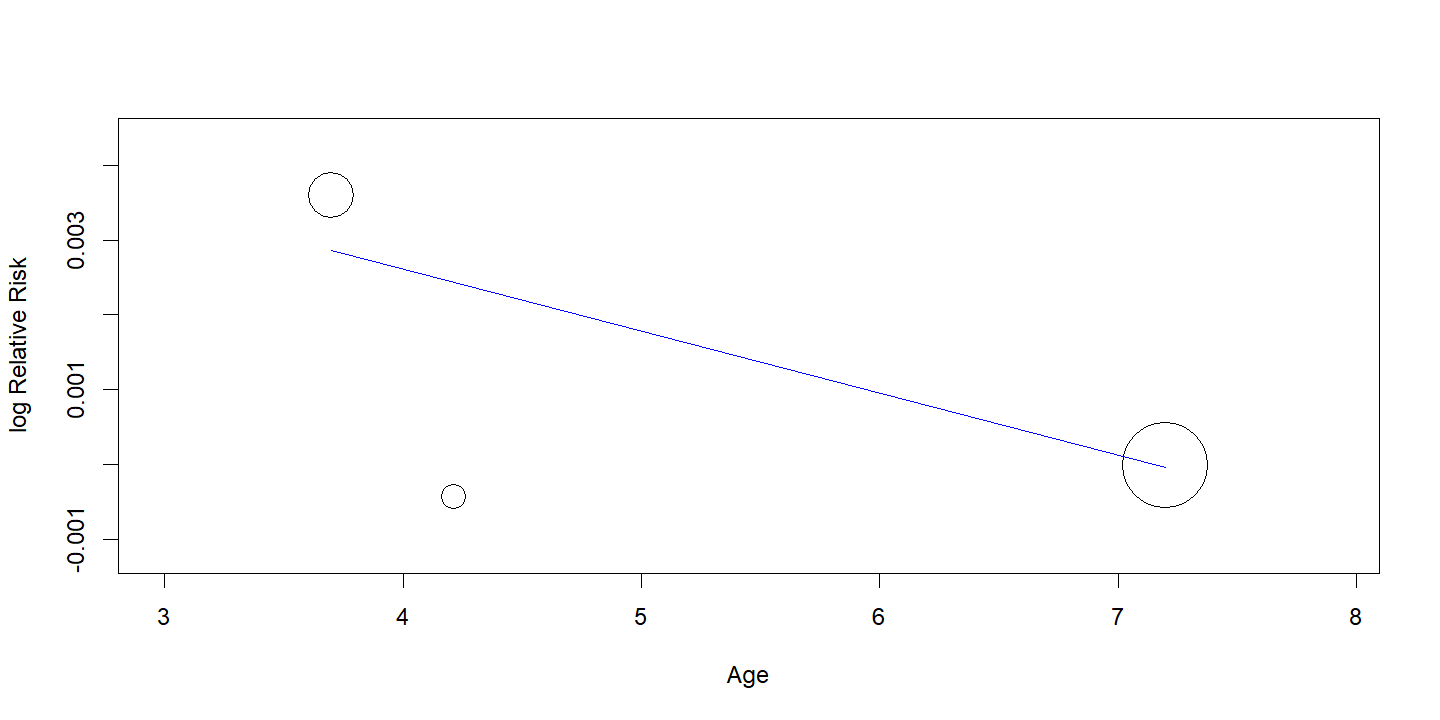


**Supplementary figure 3:** Regression plot for AEs and seroconversion rate between Healive® and live-attenuated vaccine. This figure shows the relationship between AEs and seroconversion rate for the Healive® vaccine, with the Omnibus p-value = 0.413.
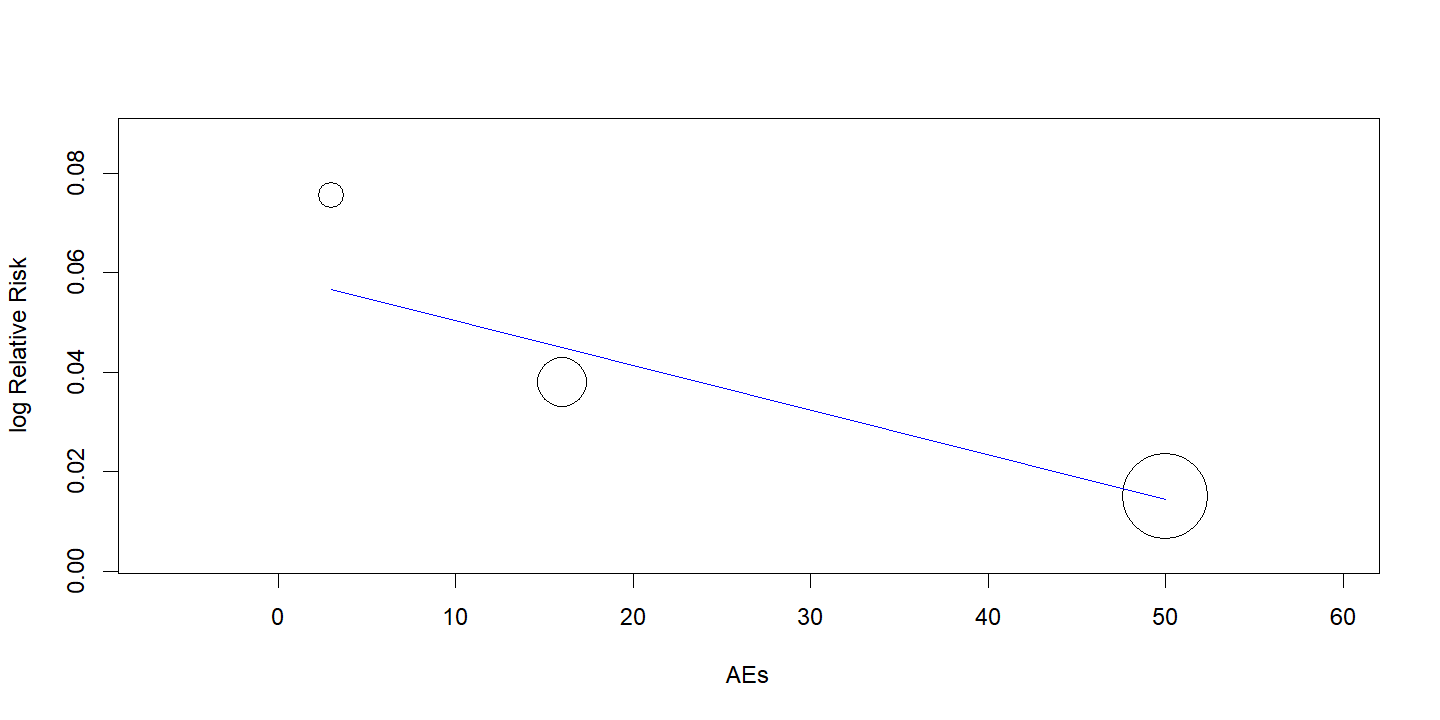


**Supplementary figure 4:** Regression plot for age and seroconversion rate between Healive® and live-attenuated vaccine. This figure shows the relationship between age and seroconversion rate for the Healive® vaccine, with the Omnibus p-value = 0.468.


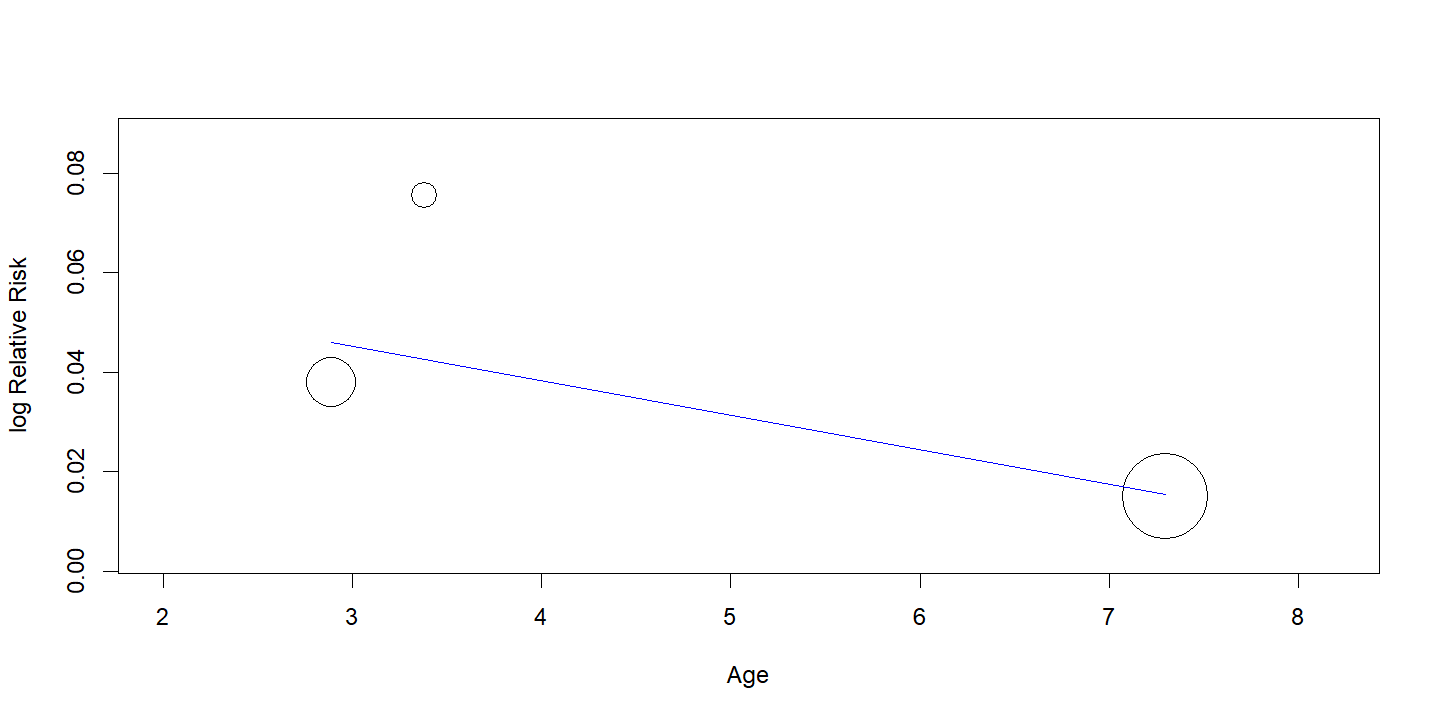

Supplement: Supplementary file 1 — Supplementary Material 1 [file 13052_2025_1996_MOESM1_ESM.docx]
